# The Effect of Substrate Type on the Optical and Structural Properties of Sol–Gel ZnO and ZnO:Ga Films

**DOI:** 10.3390/molecules30163342

**Published:** 2025-08-11

**Authors:** Tatyana Ivanova, Antoaneta Harizanova

**Affiliations:** Central Laboratory of Solar Energy and New Energy Sources, Bulgarian Academy of Sciences, Tzarigradsko chaussee 72, 1784 Sofia, Bulgaria; tonyhari@phys.bas.bg

**Keywords:** sol–gel coatings, gallium dopant, optical properties, substrate type

## Abstract

In this work, a sol–gel spin coating method was applied to obtain ZnO and ZnO:Ga thin films on a glass and ITO-coated glass substrate. Their structural, optical, and electrical properties were investigated with respect to their dependence on the different substrates, the number of layers (two and four), and the annealing temperature (300 and 400 °C). X-ray diffraction (XRD) patterns showed a hexagonal structure corresponding to the wurtzite phase for ZnO and ZnO:Ga films. ZnO films, deposited on a glass substrate, reveal greater crystallite sizes compared with ZnO films obtained from an ITO substrate. A Ga dopant worsened film crystallization. X-Ray photoelectron spectroscopy (XPS) proves the presence of Ga in a ZnO structure. ZnO films show lower transparency and haze values up to 44.12 (glass substrate) and 33.73 (ITO substrate) at a wavelength of 550 nm. The significant enhancement of ZnO film transparency is observed with Ga doping (with average transmittance in the visible spectral range above 85%, independent of the substrate used). Sheet resistance values are lower for ZnO:Ga films, and the figure of merit values are better compared with those of undoped ZnO films. Work function is studied for ZnO and ZnO:Ga films, deposited on Si, ITO, and glass substrates.

## 1. Introduction

ZnO is an n-type semiconductor based on abundant and environmentally friendly elements. ZnO materials are increasingly used as active components in various fields, including gas sensors, light-emitting diodes, solar cells, etc. [1]. Metal interstitials (Zn_i_) and oxygen vacancies (V_O_) in ZnO are reported to be responsible for the intrinsic n-type conductivity as these intrinsic defects can serve as donors [2]. The conductivity of ZnO films can be further enhanced by doping with Al, Ga, and In (Group III elements) [3]. ZnO-doped materials are investigated for their possible usage as transparent conducting electrodes as components in optoelectronic devices [3].

Undoped ZnO material is found to be too resistive for TCO applications. Intrinsic ZnO is thought to be an n-type semiconductor primarily because of donor defects such as zinc interstitials (Zn_i_) and oxygen vacancies (V_O_). Undoped nonstoichiometric ZnO thin films have unstable electrical properties because the sheet resistance of the films increases under either oxygen chemisorption or desorption [4]. Doping in ZnO leads to the appearance of shallow donor states below the ZnO conduction band minima that are ionized at room temperature to increase carrier concentration and therefore reduce electrical resistivity. The most widely used doping element is Al. ZnO:Al films present good conducting properties. However, due to the small ionic size of Al^3+^ (0.39 Å) in four coordinations, it is rather mobile, hence leading to stability issues [3,4]. Another disadvantage of using an Al dopant is that the enthalpy of formation for Al_2_O_3_ is strongly negative (−17.27 eV), and the forming electrically inactive Al_2_O_3_ secondary phase in the ZnO structure is high [4]. Doping of ZnO using Ga in particular is more effective for the stabilization of lattice systems and increases the ionicity of chemical bonds in ZnO films. On the other hand, gallium oxide (Ga_2_O_3_) possesses a less negative formation enthalpy (−11.29 eV), and Ga has a larger ionic radius (0.47 Å) compared with Al; therefore, it is potentially a more stable and efficient donor dopant [5].

It is well known that Ga doping in ZnO results in high transparency (with film transmittance above 85% or even 90% in the visible spectral range) [6]. It is reported that, due to the similar ionic radii of Ga^3+^ and Zn^2+^, Ga can substitute Zn in the oxide lattice with minimal strain [7]. Ga incorporation into ZnO nanomaterials induces an increase in oxygen vacancies, thus leading to the release of free electrons, which contribute to improve the conducting properties and to better gas sensing behavior by showing high sensitivity and fast response [8]. Ga-doped ZnO materials are also known to express enhanced sensitivity to UV light, and they can be applied in photodetector devices [9].

A distinct interesting application of ZnO films due to their n-type conductivity is transparent heterostructure solar cells [10]. Transparent solar cells actively absorb UV rays, while allowing the transmission of visible light. Transparent solar cells could turn windows and electronic devices into power generators. Other ZnO composite materials, such as ZnO/SiC, are reported to exhibit better material properties, and thus, they can be excellent candidates for numerous applications, from photocatalysis and optoelectronics to gas sensing and solar cells. [11].

Recently, we studied the effect of Ga doping concentration in sol–gel ZnO films [12]. The structural, morphological, and optical properties of five-layered films were investigated in the a previous work, the used substrates were silicon wafers and quartz substrates, and the technological conditions were very different [12]. The gallium-doped films revealed optical transparency improvement, deterioration of film crystallinity, and smoother surface morphology [12].

In this work, we further developed the study of gallium doping in ZnO films. Undoped (ZnO) and Ga-doped films were deposited by the sol–gel spin coating method on two types of substrates. This study explores the structural modifications and subsequent changes in optical and electrical properties induced by a Ga additive. In addition, the effect of the number of deposited layers and thermal annealings was investigated. X-ray diffraction (XRD) and UV–VIS–NIR spectroscopy were used to investigate the substrate effect (glass, ITO) on the properties of the obtained thin films. The electrical properties, such as sheet resistance and work function, of sol–gel ZnO and ZnO:Ga films were studied. The change in the work function of ZnO and ZnO:Ga films with the number of layers and annealing temperatures is discussed.

## 2. Results and Discussions

### 2.1. XRD Characterization

**Figure 1:**
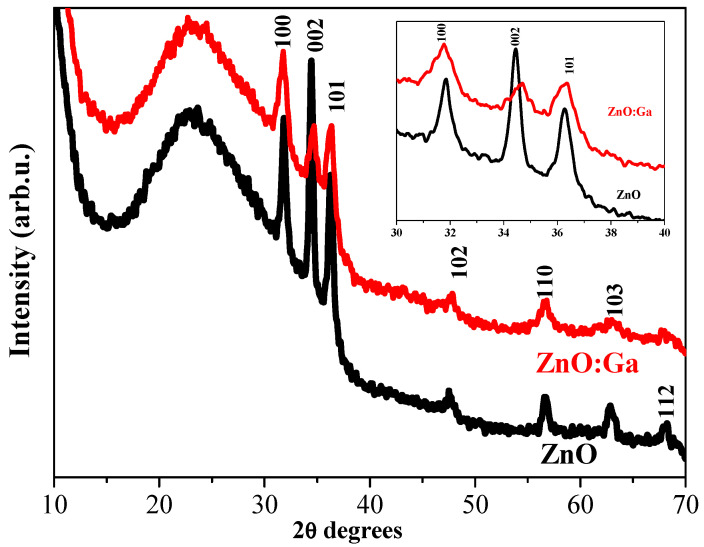
XRD patterns of sol–gel four-layered ZnO and ZnO:Ga films annealed at 400 °C. The substrate is glass. The inserted figure presents an enlarged view of the three main XRD peaks.

XRD patterns of the sol–gel ZnO and ZnO:Ga films with four layers, obtained from glass and annealed at 400 °C, are presented in Figure 1. The observed XRD lines indicate a crystallization of the films. The positions of XRD peaks correspond to the I wurtzite crystal phase (according to PDF 00-036-1451).

The recorded patterns (Figure 1), especially in the 2θ range of 30–40°, indicate that ZnO film crystallinity deteriorates due to Ga doping. The polycrystalline structure is proved for ZnO and ZnO:Ga films on glass after thermal treatment at 400 °C. In the case of ZnO film, it can be observed that a preferential orientation of the film growth along the 002 direction as the corresponding XRD line is the strongest one in the pattern. Ga doping results in suppressed growth in the 002 direction. No lines associated with Ga or Ga oxides are detected in the doped film. It is observed that the doping causes changes in the structural properties of sol–gel ZnO films on a glass substrate. Ga doping in ZnO films influences the film crystallization and the preferential crystallographic orientation.

The crystallite sizes were calculated using the Debye–Scherrer formula from the XRD data. The obtained values are presented in Table 1. The crystallite sizes of the sol–gel ZnO:Ga films are considerably smaller than those of the undoped ZnO films. A similar effect of Ga doping on the ZnO crystallization has been previously reported by others [13].

**Figure 2:**
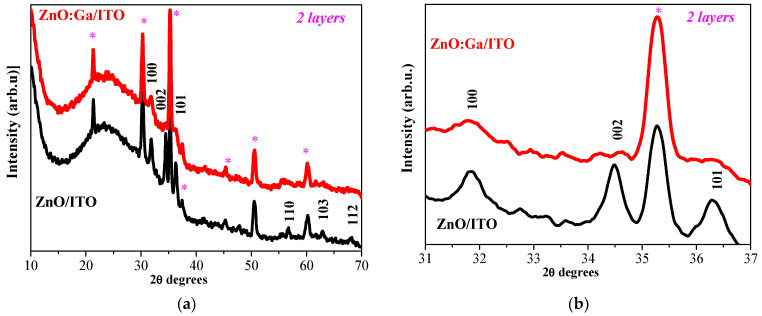
(**a**) presents XRD patterns of sol–gel two-layered ZnO (black line) and ZnO:Ga films (in red) annealed at 400 °C, and (**b**) shows an enlarged view of the three main XRD peaks. The substrate is ITO-covered glass. The asterisk marks XRD lines of ITO (PDF 01-083-3350) due to the substrate used.

XRD analysis was also performed for ZnO and ZnO:Ga films deposited on ITO substrates. The first analyzed samples were the sol–gel two-layered films annealed at 400 °C and XRD pattern given in Figure 2. The undoped ZnO film exhibits clear lines attributed to the wurtzite ZnO phase. The studied sample is well crystallized with an average crystallite size of 21.4 nm (see Table 1). The two-layered ZnO:Ga film reveals weaker XRD lines, indicating that the existence of amorphous fractions can be noticed in Figure 2b. The three strongest lines of the wurtzite phase can be identified and distinguished in the XRD pattern of the two-layered ZnO:Ga film (Figure 2b). The XRD peaks are broad and with low intensity, and they cannot be used for calculating the crystallite sizes, and the crystallite sizes are too small to be determined.

**Figure 3:**
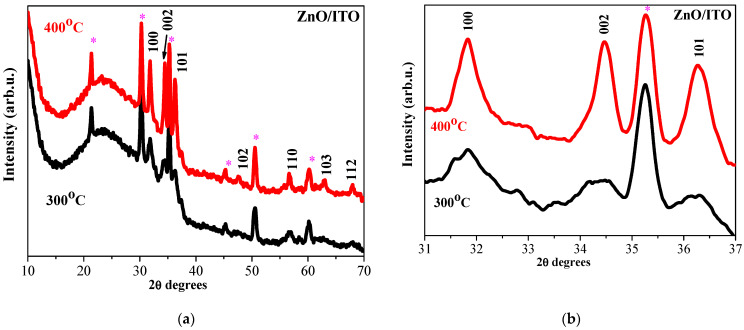
(**a**) XRD patterns of sol–gel four-layered ZnO films annealed at 300 (black color) and 400 °C (red color); (**b**) enlarged view of the three main XRD peaks in the range 31–37°. The films are deposited on an ITO substrate. The asterisk marks XRD lines of ITO (PDF 01-083-3350).

**Figure 4:**
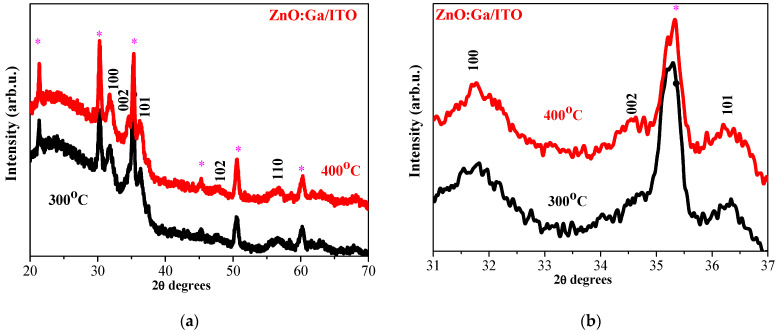
(**a**) XRD patterns of sol–gel four-layered ZnO:Ga films annealed at 300 and 400 °C; (**b**) enlarged view of the three main XRD peaks. The substrate is ITO-covered glass. The asterisk marks XRD lines of ITO coating (PDF 01-083-3350).

Increasing the number of layers induces interesting features in XRD patterns of ZnO and ZnO:Ga films deposited on ITO substrates. Figure 3 gives the XRD patterns of four-layered ZnO films annealed at 300 and 400 °C, and Figure 4 presents the XRD patterns of the corresponding thicker ZnO:Ga films.

The XRD patterns presented in Figure 3 and Figure 4 indicate that ZnO and ZnO:Ga films are crystallized in the wurtzite hexagonal phase. There are no gallium or gallium oxide phases since only peaks attributed to the hexagonal structure of ZnO are identified from XRD data. XRD analysis reveals that Ga doping causes changes in the film crystallization behavior. The sizes of the crystallites of ZnO:Ga films, obtained from ITO substrates, are reduced (Table 1).

XRD investigation reveals that Ga doping in ZnO films, deposited on glass and ITO substrates, results in suppressed and broadened XRD lines, an indication of a significant deterioration in the crystalline quality of the ZnO host. The intensity of an XRD line in the 002 direction decreases considerably compared with the line of the corresponding undoped samples. This reorientation of the crystallites is provoked by the incorporation of Ga. There are two possible reasons for this effect: the first one is that Ga substitutes Zn sites in the zinc oxide lattice, and the second is that some Ga^3+^ ions may occupy the interstitial positions in the ZnO lattice [14]. Ga doping provokes the growth of smaller crystallites.

An XRD study also exposes the substrate effect on the film crystallization. ZnO films deposited on a glass substrate possess a polycrystalline structure while forming bigger crystallites compared with ZnO films obtained from ITO substrates.

### 2.2. XPS Study

The surface composition and chemical states of ZnO and ZnO:Ga films on ITO substrates were investigated by XPS spectroscopy. The studied samples are four layered films annealed at 400 °C.

**Figure 5:**
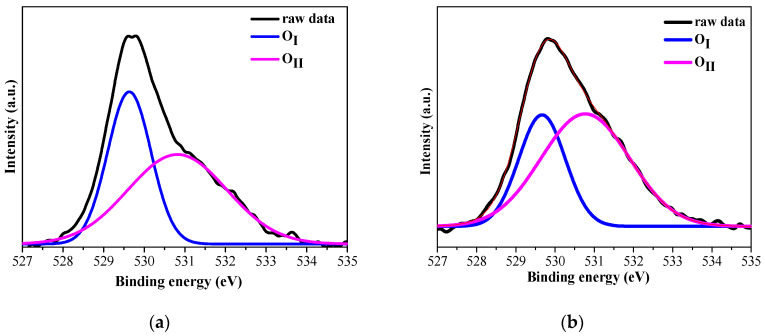
XPS spectra of O1s of (**a**) ZnO and (**b**) ZnO:Ga films deposited on ITO-coated glass and annealed at 400 °C. The colored curves correspond to the to the deconvoluted components.

Figure 5 presents the XPS core level spectra of O1s of the studied sol–gel films. The O 1s XPS spectrum of ZnO film was fitted to two contributions situated at about 529.6 eV (O_i_) and 530.8 eV (O_II_). The first peak is assigned to the O^2−^ ions on the wurtzite structure of the hexagonal Zn^2+^ ion array, which are surrounded by zinc atoms with the full supplement of nearest-neighbor O^2−^ ions. The second component at 530.8 eV is connected with O^2−^ ions in oxygen-deficient regions within the ZnO matrix [15,16]. Similarly, the asymmetric peak of O 1s of ZnO:Ga film was decomposed into two components at 529.7 (O_i_) and 530.8 eV (O_II_). The intensity ratio of O_II_/O_total_ was estimated to be 0.68 for ZnO film and 0.78 for Ga-doped ZnO film. The results indicate that oxygen vacancy sites increase with Ga doping. It is known that the n-type conductivity of ZnO is provoked by the defects and oxygen vacancies [17].

**Figure 6:**
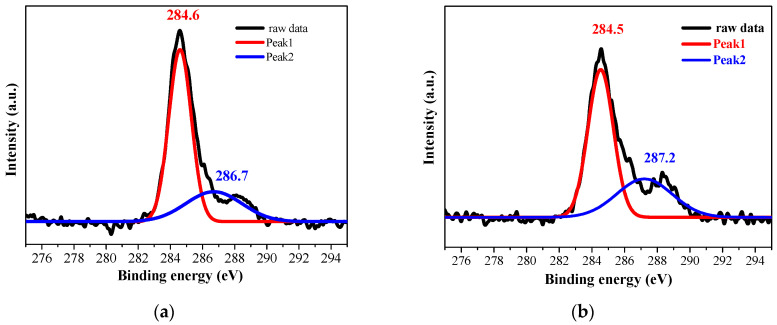
XPS spectra of C 1s of (**a**) ZnO and (**b**) ZnO:Ga films deposited on ITO-coated glass and annealed at 400 °C. The colored curves correspond to the deconvoluted components.

The C 1s spectrum was fitted with two contributions (see Figure 6). The peak at 284.5 eV is due to C–C bonding mostly due to adventitious carbon contamination. The second peak, positioned at 286.7 eV for ZnO (Figure 6a) and at 287.2 eV (Figure 6b) for ZnO:Ga, is attributed to C–O bonds probably related to surface oxidation or to the air exposure [18].

**Figure 7:**
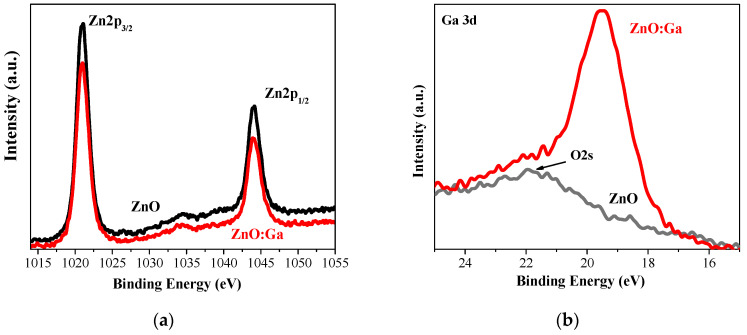
XPS spectra of (**a**) Zn 2p and (**b**) Ga 3d of ZnO (black color) and ZnO:Ga (red color) films deposited on ITO-coated glass and annealed at 400 °C.

The core-level spectra of Zn2p were analyzed in detail. The binding energy values are agreed with the literature values [15,16]. The 23.08 and 23.1 eV spin-orbit splitting for Zn 2p_1/2_ and Zn 2p_3/2_ confirms that Zn atoms are in a fully oxidized state and correspond to wurtzite ZnO [19] in ZnO and ZnO:Ga films, respectively. An XPS study clearly proves the presence of Ga in doped sol–gel films. Ga 3 d_5/2_ is located at 19.59 eV (see Figure 7b). The specific binding energy value and its position in the XPS spectrum are indicative of the chemical environment and oxidation state of gallium. A binding energy of 19.59 eV is characteristic of Ga^3+^ ions in gallium oxide and when Ga atoms are substituting Zn in the ZnO lattice [20]. It can be assigned as Ga(Zn)−O bonding [20].

The quantitative analysis of ZnO and ZnO:Ga films was carried out by using relative sensitivity factors (RSFs) based on XPS results. In ZnO film, the detected elements are C, O, and Zn, and their atomic percentages are 20.18%, 43.32%, and 36.5%, respectively. The composition of ZnO:Ga film is C, O, Zn, and Ga. The amount of carbon is 16.60%, zinc is 33.64%, oxygen is 44.26%, and Ga is 5.51%.

An XPS study confirms the wurtzite structure of the studied sol–gel films. The presence of gallium as a dopant is also proved.

### 2.3. Optical Investigation of Undoped ZnO Films on Glass and ITO Substrates

Optical properties play a great role in thin film optoelectronics and especially in transparent solar cells. Transmittance and reflectance of thin films are important properties, and they must be studied. The optical analysis is performed as a function of the number of layers and annealing temperatures. The measured film thickness values for ZnO films are 90 nm for a two-layered sample and 180 nm for a four-layered sample. The corresponding values for ZnO:Ga films are 120 nm (two-layered sample) and 240 nm (four-layered film). The film thickness is slightly changed with the annealings.

**Figure 8:**
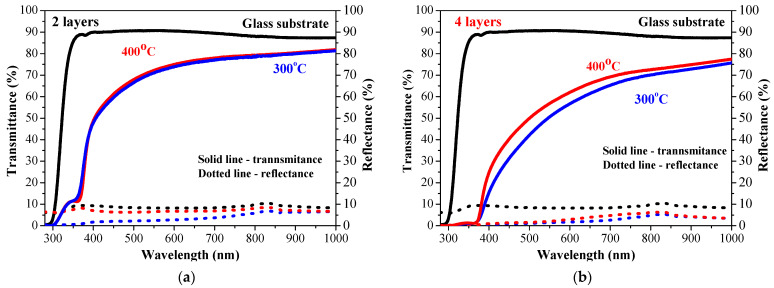
Transmittance and reflectance spectra of the sol–gel ZnO films deposited on glass substrates, where (**a**) presents two-layered samples annealed at 300 (blue curves) and 400 °C (red curves) and (**b**) presents four-layered films annealed at 300 (blue curves) and 400 °C (red curves). The spectra of a bare glass substrate (in black color) are given for comparison. The solid lines present transmittance and the dotted lines- reflectance spectra.

**Figure 9:**
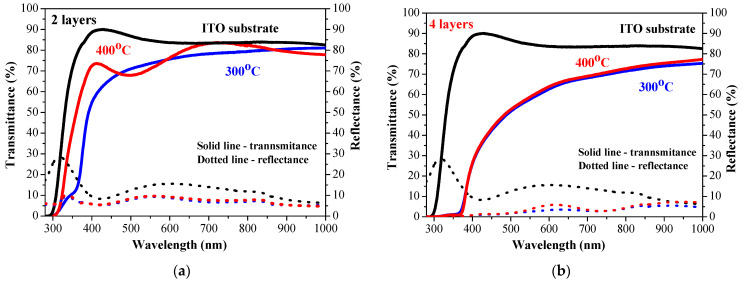
Transmittance and reflectance spectra of the sol–gel ZnO films deposited on ITO-covered glass substrates, where (**a**) is the spectra of the two-layered samples annealed at 300 (blue curves) and 400 °C (red curves) and (**b**) is the spectra of the two-layered films annealed at 300 (blue curves) and 400 °C (red curves). The spectra of a bare ITO substrate (in black color) are given for comparison. The solid lines present transmittance and the dotted lines- reflectance spectra.

Figure 8 presents the transmittance and reflectance spectra of undoped two-layered ZnO films (Figure 8a) and four-layered ZnO films obtained from glass substrates. The reflectance of the films is below 10%. The transparency is slightly decreased by increasing the number of layers. This is attributed to the fact that absorption is closely related to thickness—absorption diminished exponentially as the film thickness decreased, and thus, transparency was improved. The same tendency is observed for ZnO films obtained from ITO (see Figure 9). ZnO films deposited on an ITO substrate possess transmittance change from 74% (two layers, Figure 9a) to 55% (four layers, Figure 9b) at a wavelength of 550 nm. Thicker films (four layers) on glass reveal negligible change in transparency by increasing the annealing temperature from 300 to 400 °C, as shown in Figure 8a. The two-layered ZnO films on ITO treated at 400 °C show a transmittance (at a wavelength of 550 nm) of 74.5% similar to 74.0% of a 300 °C annealed sample (Figure 9a). Two-layered ZnO films, annealed at a higher temperature, show an interference spectrum, an indication of better film crystallinity. An XRD study confirms this statement.

The diffuse transmittance and total transmittance spectra were recorded with a UV–VIS–NIR spectrophotometer utilizing the integrating sphere attachment. The parameter haze can be determined from these measurements [21]. The haze parameter defines the degree to which light is scattered when passing through a transparent material due to internal or surface imperfections.

The haze can be determined from the relation using the measured spectra of total (*T_total_*) and diffuse transmittance (*T_diffuse_*) [22], as follows:

(1) $$Haze=\frac{T_{diffuse}}{T_{total}}\times 100\%$$

The haze parameter is important as it measures the amount of the diffused or scattered light when passing through a transparent thin film. It is used as quality control in industry, such as in glass and transparent materials. Haze is also a significant factor in solar cell design. For example, for silicon solar cells, TCO (transparent conductive oxide) films on the cell front side with high haze (*H*) can effectively scatter sunlight, lengthen the light traveling path, increase the photon absorption, and then enhance the short-circuit current density (Jsc) and the energy conversion efficiency of the solar cells [23]. Materials with high haze tend to possess low transmittance.

**Figure 10:**
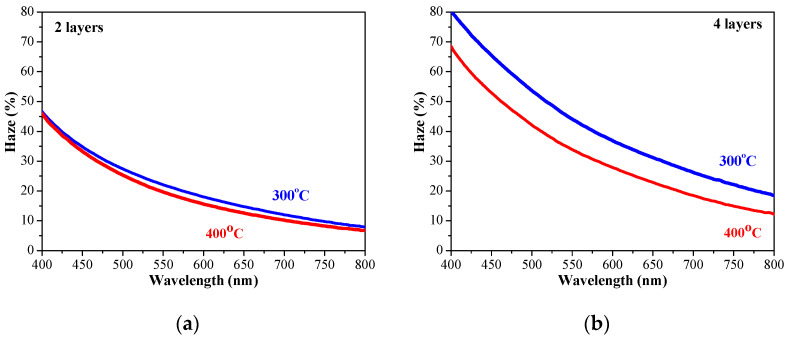

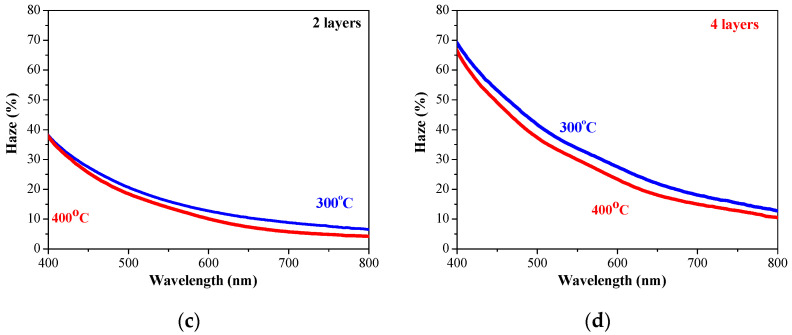
Spectral dependence of haze values of sol–gel ZnO films (**a**,**b**) with two and four layers obtained from a glass substrate and (**c**,**d**) haze values of two- and four-layered ZnO films deposited on ITO-covered glass. The films are annealed at 300 (blue lines) and 400 °C (red lines).

The determined values of the haze parameter of sol–gel ZnO films obtained from glass and ITO substrates are given in Figure 10.

Table 2 presents the average transmittance and average reflectance values (estimated for the spectral range 450–700 nm), the optical band gap, and the haze values. It reveals interesting features. ZnO films obtained from glass substrates show lower transmittance and higher haze values compared with ZnO films on ITO despite the fact that an ITO substrate has lower transmittance compared with the glass transparency.

This can be due to a rough film surface. An XRD study and the data presented in Table 1 exhibit that the microstructure of ZnO film, obtained from a glass substrate, is with a polycrystalline nature, and the crystallites are with an average size of 20.9 nm. ZnO films deposited on an ITO substrate at the same technological conditions also have a polycrystalline structure, but the grown crystallites are considerably smaller with an average size of 10.7 nm. This suggests that ZnO films on ITO substrates grow smoother and respectively manifest better transparency.

The haze values of ZnO films are high. The obtained optical data, including the haze values and the transmittance values, indicate that the optical properties of ZnO films must be improved in order to use these films in optoelectronic devices and transparent solar cells. A good strategy to achieve enhanced optical properties is to use an appropriate dopant in ZnO films. Gallium (Ga) is known to be an effective doping element for improving the optical properties of ZnO.

We use the first derivative dT/dλ to estimate the band gap [24]. The optical band gap values are similar, and they seem to be not dependent on the substrate used. The values of the optical band gap for ZnO films are in the frames reported in the literature [25].

### 2.4. Optical Investigation of Gallium-Doped ZnO Films on Glass and ITO Substrates

**Figure 11:**
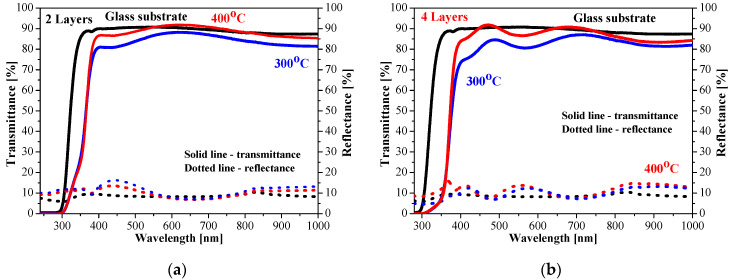
Transmittance and reflectance spectra of sol–gel ZnO:Ga films with (**a**) two and (**b**) four layers obtained on glass substrates. The films are annealed at 300 (blue lines) and 400 °C (red lines). The transmittance and reflectance spectra (in black color) of a bare glass substrate are given for comparison.

**Figure 12:**
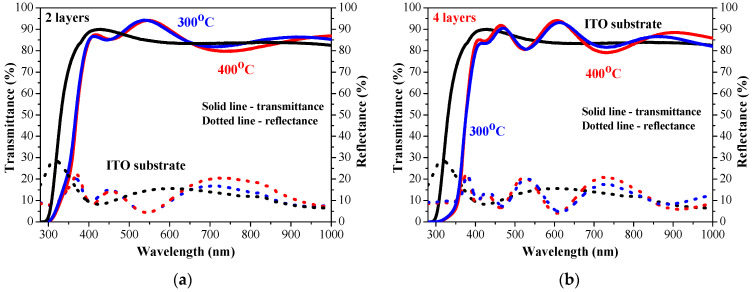
Transmittance and reflectance spectra of sol–gel ZnO:Ga films with (**a**) two and (**b**) four layers obtained from ITO-covered glass substrates. The films are annealed at 300 (blue lines) and 400 °C (red lines). The transmittance and reflectance spectra (in black color) of a bare ITO substrate are given for comparison. The solid lines present transmittance and the dotted lines- reflectance spectra.

The optical transparency of sol–gel ZnO:Ga films is excellent, both on glass and ITO substrates (see Figure 11 and Figure 12). Their transmittance is very high, and it almost approaches the transmittance values of the corresponding bare substrate (the spectra of uncoated substrates are presented). This proves that gallium doping considerably improves the optical properties. This is also an indirect proof that these thin films possess uniform and smooth surfaces. A previous FESEM and AFM study [12] of five-layered ZnO:Ga films deposited on silicon wafers revealed that the increase in the gallium doping concentration in Zn solution resulted in a smoother and more uniform surface morphology of the deposited ZnO:Ga films. The film roughness was significantly diminished up to 1 nm [12].

The measured direct and total transmittance spectra are similar, and the diffuse transmittance is very low. This means that the haze values of the sol–gel ZnO:Ga films are very low. The transmittance spectra suggest that there is almost no light scattering. ZnO:Ga films deposited on glass substrates show a slight increase in transparency after 400 °C annealing (Figure 11).

The average transmittance values and the average reflectance values estimated for the spectral range 450–700 nm are given in Table 3, together with the calculated optical band gap values of ZnO:Ga films. The first derivative of the transmittance spectrum was used for the determination of the optical band gap.

The obtained results imply that the optical transmittance of ZnO:Ga films is slightly affected by the number of layers and the annealing procedures. The film transparency of two-layered ZnO:Ga coating on a glass substrate is changed from 85.2 to 89.7% by increasing the annealing temperature The four-layered doped films show an increase in the average transmittance values up to 88.8% after 400 °C treatment.

The film transparency is very high for ZnO:Ga films deposited on ITO substrates. The average transmittance values are comparable, regardless the film thickness difference (number of layers) and the annealing temperatures of 300 and 400 °C. This is indirect proof that the Ga-doped ZnO coatings possess uniform and smooth film morphology as there is almost no light scattering.

The comparison of the average transmittance values (Table 2 and Table 3) of undoped ZnO and ZnO:Ga films proves that Ga incorporation rapidly improves the optical film transparency. For the case of two-layered samples, this transmittance jump is approximately 15% higher. The four-layered ZnO:Ga films are significantly transparent than the ZnO film. The average transmittance value of 400 °C annealed ZnO film on an ITO substrate is 55.88%, the corresponding value of ZnO:Ga film is 87.09%, and the transparency difference is roughly 32%. A Ga dopant enhances the optical transparency of ZnO films in visible spectral range, suggesting that these films can be an excellent candidate for usage in transparent photovoltaics.

An optical band gap depends on crystallization, crystallite size, impurity presence, grain boundaries and defects, lattice strain, and stress [26]. The optical band gap of ZnO:Ga films is narrowing by increasing the number of layers and the annealing temperature, as shown in Table 3. On the other hand, the optical band gap values of Ga-doped films are greater than those of undoped ZnO films. One possible explanation for this widening with Ga doping can be the Burstein–Moss (B-M) effect, which is induced by Ga^3+^ doping [27]. The B–M effect suggests that the optical band gap becomes broader due to the increase in the carrier concentration in the doped metal oxides [28,29]. Another possible reason for the optical band gap widening of Ga-doped ZnO films is their smaller crystallite sizes. The quantum size can shift the optical band gap to higher energy [30]. An XRD study confirms that conclusion as it proves that a Ga dopant decreases the film crystallinity and the crystallite sizes of ZnO films.

### 2.5. Electrical Properties of Sol–Gel ZnO and ZnO:Ga Films

The sheet resistance values were measured by a four-probe method of the thin films deposited on glass substrates. The obtained results are given in Table 4. ZnO films possess higher sheet resistance values by increasing the annealing temperature. The doped films exhibit lower values, proving that the film conductivity is improved with gallium addition.

The requirements for transparent conductive metal oxides in solar cells include two competing and important factors: optical transparency and sheet resistance. The transmittance affects the charge generation within solar cells. Low sheet resistance can improve device efficiency [31,32]. The figure of merit of Haacke (*FOM*) is defined as the relationship of the material transparency and the measured sheet resistance. Thus, it allows for comparing different types of transparent conducting materials as *FOM* determines their effectiveness for a specific application. Figure of merit was determined from the given relation [33], as follows:

(2) $$FOM=\frac{T_{average}^{10}}{R_{sheet}}$$

where *T_average_* is the average transmittance (the values were taken from Table 2 and Table 3, and they are estimated for the spectral range 450–700 nm), and *R_sheet_* is the measured sheet resistance by the four-point probe. The obtained values of ZnO and ZnO:Ga films deposited on glass substrates are given in Table 4. The results show that the *FOM* values are dependent on Ga depending on doping as *FOM* values are better and higher for sol–gel ZnO:Ga films.

A higher *FOM* value indicates better film quality and a good balance between electrical conductivity and optical transparency at a particular wavelength or across a range of wavelengths in the optical spectrum. These values are comparable to reported *FOM* values of Al-doped ZnO thin films ranging from 10^−4^ to 10^−2^ Ω ^−1^ [33]. The *FOM* values from 11 × 10^−3^ to 5.6 × 10^−3^ Ω^−1^ were determined for Ga-doped ZnO films deposited by an atmospheric pressure plasma jet [34].

Work function is defined as the energy needed for removing an electron from the Fermi energy to the vacuum level [35]. The work function is not a constant of a certain material as it can be modified. The WF of materials is especially relevant for photocatalysis and solar cells as it controls the band alignments of interfaces such as metal oxide/metal contacts forming Schottky barriers and n-type metal oxide/p-type semiconductor interfaces forming p-n junctions. [36,37]. By tuning the work function of metal oxide layers, the performance of a heterojunction solar cell can be improved. Matching the WFs of different layers in a solar cell is crucial for efficient charge separation and transport, whereas in the case of photocatalysis, it influences the redox potentials and charge separation, impacting the overall reaction rates [38]. Work function (WF) establishment is important for ZnO thin film applications.

The WF values are affected by film thickness, the preparation method, thermal treatments, UV excitation, and doping [35,39]. The work function is measured with a Scanning Kelvin Probe, and the obtained results are shown in Table 5. It is known that the work function of bulk ZnO is around 4.45 eV [40].

Higher WF values are found for ZnO and ZnO:Ga films deposited on ITO substrates. It is also established that WF values become greater by increasing the annealing temperature. The sol–gel thin films manifest higher values compared with bulk ZnO, and this can be associated with quantum confinement effects due to particle nanosizes.

Tuning the work function can provide an effective application of transparent conductive oxide. Some authors claim that the work function of ZnO and doped ZnO films decreases when oxygen vacancies are filled, and respectively, the increment in oxygen vacancies results in larger WF values [6]. In our case, ZnO:Ga films possess smaller WF than the corresponding ZnO films. The reported WF values of ZnO and Ga-doped ZnO films are in the wide range of 3.3 to 5.2 eV [38,41,42,43].

## 3. Materials and Methods

The detailed preparation of Zn sol solution was already reported [12]. The zinc precursor was zinc acetate dihydrate (Zn(CH_3_COO)_2_.2H_2_O, Riedel-de Haen, Hannover, Germany) dissolved in absolute ethanol (C_2_H_5_OH), Merck KGaA, Darmstadt, Germany, absolute for analysis). Monoethanolamine (MEA, Fluka AG, Buchs, Switzerland, 98%) was used as the complexing agent at a MEA/Zn molar ratio equal to 1 [11]. The obtained 0.4 M Zn sol was translucent with no sedimentation. Ga-mixed Zn sol was developed by dissolving 4 wt% of gallium (III) nitrate hydrate (GaN_3_O_9_.xH_2_O, Sigma-Aldrich, Saint Lous, USA) into a proper Zn sol volume. Zn and mixed Zn–Ga sols were stirred at 40 °C/2 h by using a magnetic stirrer (ARE, Velp Scientifica s.r.l., Usmate, Italy). After that, the two sols were ultrasonically processed at 45 °C/2 h in ultrasonic bath (ultrasonic cleaner, UST 2.8-100, Siel Ltd., Gabrovo, Bulgaria). The obtained sol solutions were found to be translucent and sediment-free.

ZnO and ZnO:Ga films were spin-coated (employing the spin coater system: spin coater P 6708, PI-KEM Limited, Staffordshire, UK) on preliminary cleaned substrates. The used substrates were glass and ITO glass (1.1 mm thick, unpatterned, Ossila Ltd., Sheffield, UK). The cleaning procedure of the substrates included several steps: The first step was sonication for several minutes in 1% Hellmanex III (Ossila Ltd., Sheffield, UK) solution at a temperature of 70 °C. Then the substrates were rinsed carefully twice in hot deionized water. Finally, the substrates were held in a UV ozone cleaner (Ossila type, Ossila Ltd., Sheffield, UK) for 15 min.

The substrates were rotated at a speed of 2000 rpm/30 sec after a certain amount of the sol solution was applied on the substrate. After the spin coating process, the films were heated at 300 °C for 10 min in a furnace (preheating temperature treatment). The preheating temperature is a key factor affecting solvent vaporization, decomposition of the precursor material, and crystallization evolution. The coating and preheating procedures were repeated two and four times to fabricate ZnO and ZnO:Ga films with different thicknesses. High temperature annealings were performed at 300 and 400 °C for 1 h in ambient air with a controlled constant heating and cooling rate of 10 °C/minute of the furnace (TOKMET-TK Ltd., Varna, Bulgaria). This technological sequence resulted in ZnO and Ga-doped ZnO thin films with good quality, uniformity, and homogeneity. The thickness of sol–gel ZnO and ZnO:Ga films was determined by an LEF 3 M laser ellipsometer supplied with He–Ne laser operating at a 638.2 nm wavelength. The film thickness measurements were performed for the films deposited on glass substrates. The measured thickness values for ZnO films were 90 nm for a two-layered sample and 180 nm for a four-layered ZnO. The corresponding values for ZnO:Ga films were 120 nm (two-layered sample) and 240 nm (four-layered film). The thickness was negligibly changed with the thermal treatments.

The structural properties and the crystallinity of ZnO and ZnO:Ga coatings were examined by the XRD method (Bruker D8 Advanced diffractometer, Bruker AXS GmbH, Karlsruhe, Germany) with CuKα radiation in the scanning range 2θ = 10–90° and a constant rate of 0.02. s^−1^. Optical spectra of the deposited films on glass and ITO substrates were measured with a UV–VIS–NIR Shimadzu 3600 double-beam spectrophotometer (Shimadzu Corporation, Kyoto, Japan) in the spectral range of 240 nm to 1800 nm. The instrument resolution was 0.1 nm. The direct transmittance spectra were taken against air as a reference. The reflectance spectra were measured with a specular reflectance accessory (5° incidence angle), and the reference was an Al-coated mirror. The total and diffuse transmittance spectra were recorded using a UV–VIS–NIR Shimadzu 3600 double-beam spectrophotometer equipped with an integrating sphere attachment (the integrating sphere was coated with barium sulfate (BaSO_4_), and it had a 60 mm diameter). The sheet resistance of sol–gel films obtained on the glass substrates was determined by using a four-point probe method (model FPP-100VEECO instrument). Work function measurements were performed with the Scanning Kelvin Probe microscope SKP5050 (KP Technology, Wick, Scotland) with a resolution of 1–3 meV, a PC-controlled microscope with off null with parasitic capacity rejection and a 3D map of surface potential visualization.

X-ray photoelectron spectroscopy was used for determining the oxidation states of ZnO and ZnO:Ga films. The XPS measurements were performed with a Kratos AXIS Supra spectrometer (Kratos Analytical Limited, Manchester, UK). The achromatic Al X-ray source under a vacuum better than 10^−8^ Pa at a 90 degree take-off angle was used. The survey scan was performed in the energy range of 0 to 1200 eV, and the other parameters were a pass energy of 160 eV and a step of 0.5 eV with 1 sweep. A high-resolution analysis was accomplished by increasing the number of sweeps and by lowering the pass energy to 20 eV at steps of 100 meV. The energy calibration of the taken spectra was performed by the C1s photoelectron line at 284.6 eV. The surface atomic composition was determined by integrating the peak areas of the corresponding C 1s, O 1s, Ga 3d, and Zn 2p photoelectron peaks. Scofield’s sensitivity factors were used for the composition determination.

## 4. Conclusions

ZnO and Ga-doped ZnO thin films were successfully deposited by the sol–gel spin coating method on glass and ITO substrates. Their structural, optical, and electrical properties were studied depending on the used substrate and the thermal treatments. Sol–gel ZnO films deposited on a glass substrate possess bigger crystallites with respect to ZnO films obtained on an ITO substrate. An XRD study reveals that gallium doping provokes the deterioration of film crystallization and the growth of smaller crystallites. XRD and XPS analyses demonstrate that the sol–gel films crystallized in the wurtzite phase and a gallium dopant preserves the hexagonal ZnO structure. XPS spectroscopy confirms the presence of gallium. The effect of the annealing temperatures at 300 and 400 °C on the transmittance, reflectance, and optical band gap is monitored for ZnO and Ga-doped ZnO thin films deposited on glass and ITO substrates. Optical transparency is greatly enhanced with the introduction of gallium. ZnO:Ga films possess a larger optical band gap than that of ZnO films. A gallium dopant improves sheet resistance and the figure of merit values. The effect of substrate type (glass, ITO, Si) on the work function is investigated. Based on the obtained results of the optical and electrical properties, we can propose that ZnO:Ga thin films possess promising properties for applications as transparent conductive oxides and in transparent solar cells.

## Figures and Tables

**Table 1:** The calculated values of the crystallite sizes of sol–gel ZnO and ZnO:Ga films deposited on glass and ITO substrates. The sizes were estimated from XRD data focusing on the three main lines in the diffraction patterns assigned to wurtzite ZnO.

Material	Substrate	Number of Layers	Annealing	XRD Line Positions	XRD Plane	Crystallite Size [nm]
ZnO	Glass	4	400 °C	31.82	100	21.64
				34.45	002	24.77
				36.29	101	16.36
ZnO	ITO	4	300 °C	31.81	100	4.30
				34.29	002	6.70
				36.13	101	5.16
ZnO	ITO	2	400 °C	31.84	002	25.24
				34.49	100	20.95
				36.29	101	17.93
ZnO	ITO	4	400 °C	31.78	100	3.40
				34.46	002	15.85
				36.28	101	12.84
ZnO:Ga	Glass	4	400 °C	31.37	100	2.54
				34.65	002	7.81
				36.27	101	8.14
ZnO:Ga	ITO	4	300 °C	31.74	100	Broad peak
				34.80	002	No clear peak
				36.34	101	Broad line
ZnO:Ga	ITO	2	400 °C	31.62	100	4.54
				34.80	002	5.20
				36.34	101	9.99
ZnO:Ga	ITO	4	400 °C	31.71	100	4.96
				34.81	002	4.27
				36.39	101	13.58

**Table 2:** Average transmittance values (*T_average_*), average reflectance values (*R_average_*) (estimated for the spectral range 450–700 nm), optical band gap (*E_g_*), and haze values of sol–gel-derived ZnO films deposited on glass and ITO substrates.

Substrate	Number of Layers	Annealing	*T_average_* (%) 450–700 nm	*R_average_* (%) 450–700 nm	*E_g_* (eV)	Haze 550 nm
Glass *	*2*	300 °C	68.41	2.55	3.29	22.10
		400 °C	69.64	2.97	3.27	25.33
	**4**	300 °C	47.31	1.47	3.20	44.12
		400 °C	53.95	2.46	3.195	33.87
**ITO** **	2	300 °C	71.95	7.53	3.28	16.00
		400 °C	71.67	2.34	3.27	13.90
	4	300 °C	54.96	8.06	3.20	33.73
		400 °C	55.88	3.36	3.19	29.93

* *T_average_* = 90.32%, *R_average_* = 8.44%; ** *T_average_* = 85.72%, *R_average_* = 13.02%.

**Table 3:** Average transmittance values (*T_average_*), average reflectance values (*R_average_*) (estimated for the spectral range 450–700 nm), and optical band gap (*E_g_*) of sol–gel-derived Ga-doped ZnO films deposited on glass and ITO substrates.

Substrate	Number of Layers	Annealing	*T_average_* [%] 450–700 nm	*R_average_* [%] 450–700 nm	*E_g_* [eV]
Glass *	*2*	300 °C	85.28	10.91	3.39
		400 °C	89.71	9.68	3.37
	**4**	300 °C	82.13	10.14	3.34
		400 °C	88.80	10.52	3.33
ITO **	2	300 °C	87.89	10.9	3.39
		400 °C	87.82	11.32	3.36
	4	300 °C	86.87	11.76	3.33
		400 °C	87.09	11.93	3.32

* *T_average_* = 90.32%, *R_average_* = 8.44%; ** *T_average_* = 85.72%, *R_average_* = 13.02%.

**Table 4:** Sheet resistance and *FOM* values of undoped ZnO and ZnO:Ga films on a glass substrate.

Material	Number of Layers	Annealing	Sheet Resistance (Ω/□)	*FOM* (Ω^−1^)
**ZnO**	2	300 °C	252	0.89 × 10^−4^
		400 °C	380	0.71 × 10^−4^
	4	300 °C	222	0.25 × 10^−5^
		400 °C	369	0.57 × 10^−5^
**ZnO:Ga**	2	300 °C	189	1.1 × 10^−3^
		400 °C	253	1.3 × 10^−3^
	2	300 °C	100	1.4 × 10^−3^
		400 °C	125	2.4 × 10^−3^

**Table 5:** Work function values of the obtained sol–gel films deposited on Si wafers, glass, and ITO substrates.

Substrate	Number of Layers	Annealing	ZnO Films WF [eV]	ZnO:Ga Films WF [eV]
**Si**	2	300 °C	4.86	4.66
		400 °C	4.86	4.69
	4	300 °C	4.83	4.65
		400 °C	4.86	4.68
**glass**	*2*	300 °C	4.37	4.65
		400 °C	4.56	4.64
	**4**	300 °C	4.61	4.62
		400 °C	4.71	4.65
**ITO**	**2**	300 °C	4.72	4.67
		400 °C	4.92	4.72
	4	300 °C	4.86	4.89
		400 °C	4.91	4.90

## Data Availability

Data are contained within the article.
